# Attentional Refreshing in the Absence of Long-Term Memory Content: Role of Short-Term and Long-Term Consolidation

**DOI:** 10.5334/joc.246

**Published:** 2023-01-11

**Authors:** Maximilien Labaronne, Gabriel Jarjat, Gaën Plancher

**Affiliations:** 1 Université Lumière Lyon 2, Laboratoire d’Etude des Mécanismes Cognitifs, Bron, France; 2 Centre de Recherches sur la Cognition et l’Apprentissage, Université de Tours, France; 3 Institut Universitaire de France (IUF), France

**Keywords:** working memory, long-term memory, memory

## Abstract

Contradictory results in the literature suggest that attentional refreshing can seemingly not operate efficiently in the absence of semantic representations, while at the same time it does not rely directly on retrieval from semantic memory. The objective of the present study was a better understanding of the bidirectional links between working memory (WM) and long-term memory (LTM), by assessing on the one hand the role of WM mechanisms in long-term recall and on the other hand how their functioning is modulated by the prior LTM content. Through two experiments, we investigated a new hypothesis: attentional refreshing requires stable WM representations independently of the presence or the absence of associated LTM traces. We manipulated this stability through short-term consolidation (Experiment 1) and multiple presentations of memoranda (Experiment 2) to evaluate how it would affect maintenance of words and pseudowords. While we found that lexicality, short-term consolidation and multiple presentations affected short-term and long-term recall, both experiments converged on the conclusion that none of these factors modulated the effect of the cognitive load of the concurrent processing task, suggesting that refreshing does not depend on LTM content nor WM representations’ stability. Additionally, we found that delayed recall performance was not affected by the cognitive load, in contradiction with previous literature. These results provide new insight into the functioning of refreshing and the links between WM and LTM.

## Introduction

Working memory (WM) is generally defined as a capacity-limited system underpinning cognitive tasks through the short-term maintenance and manipulation of information (Baddeley & Hitch, 1974). Over the years, researchers have become increasingly interested in the relationship between WM and long-term memory (LTM). In this regard, many studies have focused on investigating the long-term effects of one WM maintenance mechanism, *attentional refreshing* (Camos & Portrat, 2015; Jarjat et al., 2018; Loaiza & McCabe, 2012, 2013). Nevertheless, the functioning of refreshing and its relation to LTM are still far from being fully understood. The aim of the present study is to clarify the bidirectional relation between WM and LTM, and more precisely what is the relation between attentional refreshing and semantic content in LTM.

### Working memory maintenance and long-term retention

The WM literature mainly distinguishes two maintenance mechanisms for verbal information: verbal rehearsal and attentional refreshing (Camos, 2015). It is well known that verbal information can be maintained through a silent repetition, named *articulatory rehearsal, subvocal repetition* or *verbal rehearsal* (Atkinson & Shiffrin, 1968; Baddeley et al., 1975; Belleville et al., 1992). The role of attention for WM functioning is now clearly established and a second maintenance mechanism, called *attentional refreshing*, has been extensively studied. Attentional refreshing is defined as a domain-general maintenance mechanism relying on attentional resources to keep information active in WM (Barrouillet et al., 2004), that is thought to be distinct from verbal rehearsal (Camos, 2015). Through a brief attentional focus on a WM representation, this process would increase the activation level of information recently presented, encoded or retrieved to keep it in an accessible state (for a review, see Camos, Johnson, et al., 2018).

Studies on refreshing mainly rely on the use of complex span tasks. While simple span tasks only require maintenance of items, in complex span tasks memoranda are presented sequentially and separated by a concurrent processing activity that forces a constant attentional switching between maintenance and processing. Compared to simple span tasks, complex span tasks impair immediate recall performance but improve delayed recall, and this effect is greater for first-presented items than last-presented items (McCabe, 2008). As it is thought that refreshing takes place during free time between every processing activity, these results suggest that the performance at delayed recall increases with the number of refreshing opportunities. In line with this idea, it has been observed that the effect of complex span tasks at delayed recall is not driven by the retrieval demand at immediate recall (Loaiza et al., 2020), and that increasing the number of processed items between each memoranda leads to better episodic memory performance (Loaiza & McCabe, 2012). The Time-Based Resource-Sharing (TBRS) model proposes that the refreshing availability is modulated by the cognitive load of the concurrent task, which is defined as the portion of the total time of the task during which attention is diverted from maintenance (Barrouillet et al., 2004). The cognitive load can be manipulated by modulating the pace of the concurrent processing activity, its difficulty or the number of distractors, all of which vary the portion of the total time during which attention is available for maintenance. Studies have shown that increasing cognitive load reduces performance at immediate and delayed recall in complex span tasks (Barrouillet et al., 2007; Camos & Portrat, 2015; Jarjat et al., 2018; Plancher & Barrouillet, 2013). Together, these results support the idea that besides short-term retention, refreshing also promotes long-term retention.

### Impact of long-term memory representations on working memory maintenance

It is worth noting that verbal WM experiments are generally conducted using known material (i.e., letters or words). Therefore, most of the time WM tasks do not require new learning of the presented memoranda, as they are already represented in the participants’ LTM. Thus, it seems relevant to question how preexisting content of LTM affects WM maintenance by examining the WM maintenance effects on long-term retention of novel material. For instance, a study investigated whether the beneficial effect of refreshing on long-term retention could be generalized to pseudowords (Loaiza, Duperreault, et al., 2015). Results have shown that the benefit of complex over simple span task on delayed recollection was observed with words but not with pseudowords. To account for these results, the authors proposed that preexisting semantic representations may be necessary for refreshing to be optimal. Similarly, other studies suggest that refreshing cannot be used to maintain unconventional characters (Ricker & Cowan, 2010), character font (Vergauwe et al., 2014) or unfamiliar melodies (Nees et al., 2017). These results are consistent with recent WM theoretical models. The covert retrieval model (Loaiza, Duperreault, et al., 2015; McCabe, 2008) assumes that refreshing may involve covert retrieval of semantically meaningful memoranda from LTM, increasing the context-content association and facilitating their recall. The revised version of the TBRS model makes a similar prediction by including a redintegration mechanism, allowing degraded traces to be reconstructed from the content of LTM (Barrouillet & Camos, 2015). Together, this literature seems to indicate that novel material, for which there is no representation in LTM, could not be refreshed. Such material could therefore not benefit from refreshing on long-term retention, supporting the idea that this mechanism may need semantic representations to operate.

However, results from other studies conflict with this proposal. For instance, Loaiza and Camos (2018) observed that when an item is forgotten at immediate recall, it is more easily recalled after a semantic cue than after a phonological cue when refreshing was used. Yet, this effect did not interact with the cognitive load of the concurrent task, that is, with refreshing opportunities. Similarly, it has been shown that word frequency and lexicality effects, known to affect the ease of access to semantic memory, affect recall without interacting with manipulations of refreshing nor refreshing speed (Camos, Mora, et al., 2018). Together, these results challenge the idea that refreshing relies on semantic representations. To our knowledge, there is currently no proposal that reconciles the finding that refreshing is not optimal when maintaining an unknown memorandum, while at the same time it does not seem to directly rely on retrieval from semantic memory. Thus, to clarify to what extent refreshing is affected by the prior content of LTM, we considered a new hypothesis.

We proposed that refreshing might be independent of LTM. Because semantic representations are, by definition, stable representations, results in the literature could rather be explained by a confound between the presence of prior semantic content and the resulting stability of WM representations. In such a case, preexisting representations in LTM could facilitate initial processes on stimuli, increasing the stability of WM representations and allowing their refreshing. LTM would therefore play a role in constructing a refreshable WM representation, without being involved in the refreshing mechanism per se. Consequently, WM representations of unfamiliar memoranda would be too fragile to be efficiently refreshed. Thus, increasing the stability of novel representations in WM should allow their refreshing. To do so, one could rely on the use of short-term consolidation, a WM mechanism further described below.

### Short-term consolidation in working memory

Short-term consolidation is defined as the transformation of transient sensory inputs into stable WM representations, that can be manipulated and recalled later (Bayliss et al., 2015; Jolicœur & Dell’Acqua, 1998; Ricker et al., 2018). This idea originated from an effect called the attentional blink (Chun & Potter, 1995; for a more detailed historical overview, see Ricker et al., 2018) corresponding to participants’ inability to report the presence or identity of a second stimulus presented hundreds of milliseconds after the first one (Raymond et al., 1992). This effect was interpreted as reflecting the existence of a short-term consolidation mechanism based on attention (Jolicœur & Dell’Acqua, 1998), that delays consolidation of subsequent stimuli presented before its completion (Vogel & Luck, 2002). The related concept of attribute amnesia refers to the inability to recall some attributes of a recently processed stimulus (e.g., its color), even when this attribute has just been used to perform a task (Chen & Wyble, 2015). However, this effect disappears when the information has to be stored and maintained for a short delay (Chen & Wyble, 2016), suggesting that attribute amnesia could be caused by an absence of consolidation when no maintenance is required. Therefore, it appears that short-term retention cannot be achieved solely by an attentional focus, short-term consolidation being essential to its success. Experiments using complex span tasks have shown that increasing the delay between the item offset and the first distractor onset improved immediate recall performance, suggesting that allowing more time for short-term consolidation facilitates short-term retention (Bayliss et al., 2015; De Schrijver & Barrouillet, 2017; Ricker & Cowan, 2014).

As we proposed that refreshing inefficiency for novel material could be explained by the fragility of their WM representation, short-term consolidation appears to be an interesting mechanism to counteract this effect by increasing their stability. Supporting this hypothesis, it has been shown that familiar stimuli are consolidated faster than novel stimuli (Blalock, 2015), indeed suggesting that items that are not already represented in LTM could require longer consolidation times before being maintained in WM.

### The present study

There are currently conflicting results in the literature regarding the link between refreshing and semantic memory. It seems that unfamiliar memoranda cannot be refreshed efficiently (Loaiza, Duperreault, et al., 2015; Loaiza, Rhodes, et al., 2015; Nees et al., 2017; Ricker & Cowan, 2010; Vergauwe et al., 2014), while at the same time refreshing appears to be independent from semantic memory (Camos, Mora, et al., 2018; Loaiza & Camos, 2018). Thus, the objective of the present study was to address this gap by evaluating a new hypothesis concerning refreshing functioning. We hypothesized that attentional refreshing does not rely on LTM, but instead on WM representations’ stability. This could constitute a source of discrepancy in prior work, in that refreshing does not rely on semantic LTM per se but does need a stable WM representation to operate efficiently, which could be provided by semantic content in LTM, episodic LTM or short-term consolidation. Following this hypothesis, results in the literature for novel material could be explained by a combination of the absence of LTM content and insufficient short-term consolidation time, that induces an instability of their WM representation and prevents their refreshing. This hypothesis was investigated by observing if refreshing could operate on novel material after stabilizing their WM representation. We manipulated stability in two ways: by promoting short-term consolidation (experiment 1) or by repeating items multiple times (experiment 2).

In a first experiment, we manipulated the stability of WM representations by varying the amount of short-term consolidation when maintaining novel material in WM. We used a complex span task with words or pseudowords to be maintained. Cognitive load of the concurrent task was manipulated to vary refreshing availability. We also manipulated the delay between the memorandum offset and the first distractor onset in order to vary the amount of short-term consolidation. If refreshing cannot be used on novel material, as suggested by the results of previous studies, the cognitive load of the concurrent task should affect immediate and delayed recall of words but not of pseudowords. If refreshing depends on WM representation stability, as stated by our theoretical hypothesis, this cognitive load effect should appear at longer consolidation times for pseudowords. This procedure was first tested in a pilot experiment, described below, that led to slight methodological improvements in this experiment.

In a second experiment, we aimed at manipulating the stability of WM representations by varying the number of repetition of memoranda. In a complex span task using words and pseudowords, we manipulated the cognitive load of the concurrent task and the number of presentations of the to-be-learned items by repeating them through different trials. As for our first experiment, we expected a cognitive load effect on words but not pseudowords at immediate and delayed recall for items presented once. We expected an increase in delayed recall performance for items presented multiple times compared to items presented once, reflecting that multiple presentations of pseudowords created traces in LTM. Following our theoretical hypothesis, we expected that a cognitive load effect would appear for immediate and delayed recall of pseudowords presented three times, but no such cognitive load effect would occur for pseudowords presented once. This interaction was expected because repeating pseudowords should increase their WM representation stability, allowing their refreshing. The absence of this interaction would support the alternative ideas that either the role of semantic LTM content is more important to refreshing than what our hypothesis stated, or that another alternative hypothesis still needs to be considered.

## Pilot Experiment

### Method

#### Participants

40 participants (27 women) between 18 and 29 years old (*M* = 22.3, *SD* = 2.11) were recruited for this experiment. All participants were native French speakers, had normal or corrected-to-normal vision, and did not self-report any history of neurological or reading problems. The experiment was conducted in line with recommendations from Helsinki declaration, and participants provided written informed consent before taking part in the study.

#### Material and design

The experiment was developed and presented using Opensesame (Mathôt et al., 2012) on a 15” laptop. The experimental material consisted of 240 target items to recall (120 words and 120 pseudowords). Words were selected from the Lexique3 database (New et al., 2001) and were high frequency (*M* = 202.33, *SD* = 104.86, in occurrence per million) singular common nouns, four to eight letters and one to two syllables long. Pseudowords were generated from the word list using the Wuggy software (Keuleers & Brysbaert, 2010), applying the following options: match letter and subsyllabic segments length and match transition frequencies. One pseudoword was selected for each corresponding word, avoiding real words phonological equivalents, and trying to respect French orthographical coherence. The concurrent parity task consisted of 360 digits, ranging from 1 to 9, with a pseudorandom presentation order.

We manipulated the cognitive load (high vs. low) and the consolidation interval (0ms vs. 2400ms) as within-subject variables, and the item type (words vs. pseudowords) as a between-subject variable. Four different versions of the experimental task were created using two random orders, so that each memory item could be presented in every cognitive load and consolidation modality.

#### Procedure

The experiment started with a training session that was followed by the experimental task, and ended with a general delayed recall. The training session was divided into two phases. The first phase comprised 54 practice trials of the parity task without time limit. The second phase consisted of four trials of the experimental task, so that every combination of cognitive load and consolidation duration would be presented.

**Figure 1:**
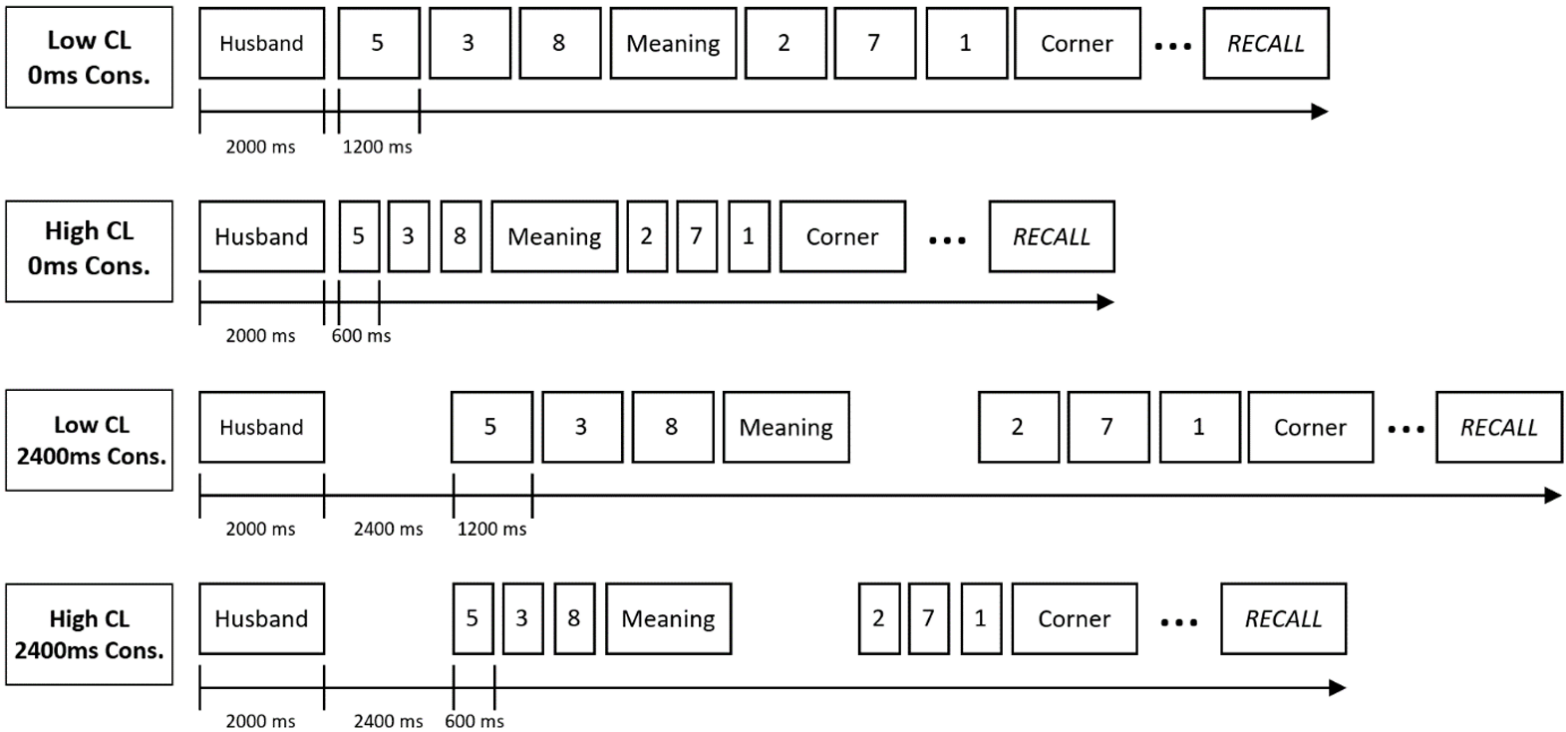
Illustration of the Four Conditions of Cognitive Load and Consolidation in the Pilot Experiment.

The experimental task was a complex span task, in which the items to be recalled could be words or pseudowords depending on the experimental group. One experimental session consisted of 24 trials of 5 memoranda. A trial started with a fixation cross presented during 1000ms, followed by the presentation of the first word or pseudoword. Each item was displayed during 2000ms and had to be read aloud (see Figure 1). Depending on the consolidation condition of the trial, the parity task was presented right after each item (0ms) or after an unfilled delay (2400ms). This task consisted of three successive digits that participants had to judge as even or odd (“m” key for even and “q” for odd on an Azerty keyboard). Digits were read silently. Depending on the cognitive load, each digit was presented for 1200ms (low cognitive load) or 600ms (high cognitive load). The presented stimuli of the trial were separated by an interstimulus interval of 250ms.

At the end of a trial, the word “*rappel*” (recall) was displayed, allowing participants to recall memory items in their original order on a response sheet provided by the experimenter. After their recall, participants had to press the spacebar key to move on the next trial, with the possibility to take breaks freely. Following the last trial, participants were required to count backward by threes from a given three-digit number for 1 min. The experimenter verified accuracy and gave immediate corrections if an error was made. After this distracting task, participants performed the delayed recall test during which they were invited to recall the 120 target items in any order. This delayed recall test stopped on participant demand or when no new item was recalled for 1 min.

## Results and discussion

To ensure that the concurrent parity task was actively processed, participants with performance lower than 70% were discarded (4 participants excluded, final sample *n* = 36). To restrain the effects of perceptive and orthographic errors, the recall score included a tolerance of one mistake on each memoranda (i.e., one addition, omission or substitution), similar to previous studies using pseudowords (Kowialiewski & Majerus, 2018; Moore et al., 2010). Main analyses at immediate recall were conducted on immediate serial scoring, which is classically used in the literature (i.e., items had to be recalled at their correct position within trials). Because we find relevant to question if the manipulated factors affected item memory or order memory, the same analyses were also conducted on immediate free scoring (i.e., evaluating only items recalled without considering their position in the trial) and compared to the effects found on immediate serial scoring. The following analyses were done using R (R Core Team, 2019) with BayesFactor (Morey et al., 2015) and bayestestR (Makowski et al., 2019) packages. We conducted Bayesian analyses of variance (BANOVA) on correct recall percentage at immediate and delayed recall, using the cognitive load (high *vs*. low), the consolidation interval (0ms *vs*. 2400ms) and the item type (words *vs*. pseudowords) as predictive variables and subjects as a random factor. Bayesian models were compared to a null model including only a random effect of subjects. The likelihood of each effect was assessed using BF_inclusion_ and BF_exclusion_, reflecting the proofs in favor or against an effect. BFs_inclusion_ were calculated across matched models (Mathôt, 2017), comparing BFs of models including the targeted effect against similar models excluding it. BFs_inclusion_ inferior to 1 are reported as BFs_exclusion_ (BF_exclusion_ = 1/BF_inclusion_) to express evidence against an effect. To interpret the resulting BFs, we referred to the following classification (Jeffreys, 1961, cited in Lee & Wagenmakers, 2013): BF at 1 shows no evidence, anecdotal evidence between 1 and 3, substantial evidence between 3 and 10, strong evidence between 10 and 30, very strong evidence between 30 and 100 and extreme evidence for BF > 100. Therefore, BFs between 1/3 and 3 were interpreted as inconclusive.

**Figure 2:**
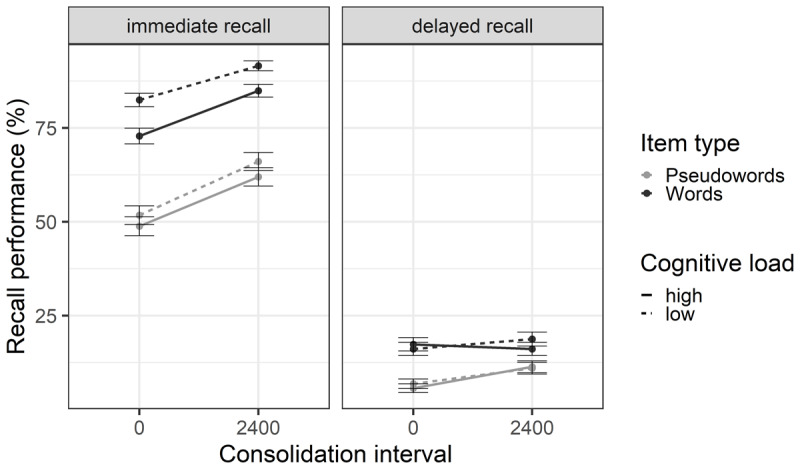
**Mean Percentage of Correct Immediate Serial Recall and Delayed Free Recall.** Percentage of correct recall is shown according to the item type (words vs. pseudowords), the cognitive load of the concurrent task (high vs. low) and the consolidation interval (0ms vs. 2400ms). The error bars represent the standard error.

At immediate recall (Figure 2), the likeliest model included the effects of item type, consolidation interval and cognitive load (BF_10_ = 3.90 × 10^16^). Recall performance was better for words (*M* = 82.95%, *SD* = 11.78) than for pseudowords (*M* = 57.16%, *SD* = 10.78), BF_inclusion_ = 1.60 × 10^5^, replicating the well-known lexicality effect (Hulme et al., 1991). As expected, the pattern of results showed extreme evidence for an effect of cognitive load (BF_inclusion_ = 194.37) with poorer recall under high (*M* = 67.79%, *SD* = 16.68) than low cognitive load (*M* = 73.75%, *SD* = 18.65), as observed in previous studies (e.g., Barrouillet et al., 2007; Plancher & Barrouillet, 2013). While we expected a cognitive load × item type interaction, evidence was inconclusive (BF_exclusion_ = 1.52). Previous results found that the advantage of complex over simple span was greater for words than for pseudowords (Loaiza, Duperreault, et al., 2015), suggesting that refreshing could not operate efficiently on pseudowords, but our results do not allow us to conclude neither for or against this interpretation. In line with previous studies (Bayliss et al., 2015; De Schrijver & Barrouillet, 2017; Ricker & Cowan, 2014), there was extreme evidence for an effect of consolidation (BF_inclusion_ = 1.44 × 10^10^) with better recall after a 2400ms delay (*M* = 76.8%, *SD* = 16.44) than without delay (*M* = 64.74%, *SD* = 18.81).

There was substantial evidence against the interaction between cognitive load and consolidation (BF_exclusion_ = 4.31), but evidence concerning the triple interaction between cognitive load, consolidation and item type was inconclusive (BF_exclusion_ = 1.80). The absence of interaction between cognitive load and consolidation duration has been observed before and interpreted as the independence of these two mechanisms (Bayliss et al., 2015) or their substitutability (De Schrijver & Barrouillet, 2017). However, we expected this interaction to be affected by the item type. Specifically, we predicted that pseudowords would benefit from longer consolidation times, allowing them to be refreshed. However, the present results do not allow us to conclude on this effect. Evidence for the consolidation × item type interaction was inconclusive (BF_exclusion_ = 2.83).

Using the immediate free recall, conclusions were the same as with the immediate serial recall, except for the consolidation × item type interaction. The likeliest model included the effects of item type, consolidation, cognitive load, and the consolidation × item type interaction (BF_10_ = 1.91 × 10^20^). There was strong evidence in favor of the consolidation × item type interaction (BF_inclusion_ = 19.25), the consolidation effect being greater for pseudowords (+12.06) than for words (+4.91). This could suggest that consolidation was more beneficial for pseudowords than for words, but only on maintaining the items themselves and not their presentation order.

At delayed recall (Figure 2), the likeliest model included the effects of item type, consolidation interval and the consolidation × item type interaction (BF_10_ = 5473.57). This model was however only 1.11 times more likely than the second-best model excluding the interaction (BF_10_ = 4926.79). As at immediate recall, there was extreme evidence for an effect of item type (BF_inclusion_ = 2248.04), with better recall performance for words (*M* = 17.11%, *SD* = 4.13) than for pseudowords (*M* = 8.73%, *SD* = 5.01). Evidence concerning the effect of consolidation interval was inconclusive (BF_inclusion_ = 2.32). The BF for the interaction between consolidation and item type allowed no conclusion on this effect (BF_inclusion_ = 1.09). Finally, contrary to our hypothesis and the literature, we found substantial evidence against an effect of cognitive load (BF_exclusion_ = 5.15). This result goes against previous literature that found a cognitive load effect at both immediate and delayed recall (Camos & Portrat, 2015; Jarjat et al., 2018). This could indicate either that refreshing failed to promote long-term learning in our experiment, raising the question of what conditions allow a refreshing benefit on LTM, or that cognitive load can sometimes affect another uncontrolled mechanism that is responsible for its effect on delayed recall. For example, recent studies suggest that refreshing only affects immediate recall while elaboration, the enrichment of an item’s representation by linking it to the already existing long-term representations (Craik & Lockhart, 1972), increases performance at delayed recall (Bartsch et al., 2018, 2019). However, replications in our next experiments will be needed to further discuss this point.

While this experiment gives a first glimpse at the expected effects, it has some limitations. First, it appears that we lacked sufficient statistical power to conclude confidently on every effect of interest. Next, performance at delayed recall was low, which may have further limited the observation of potential effects. To prevent this, a blocked design with multiple delayed recalls could have been used. Therefore, we conducted two subsequent experiments, aiming at (1) testing again the effect of short-term consolidation on WM maintenance for novel material with increased statistical power and (2) complement the investigation of our theoretical hypothesis by assessing the effect of a non-semantic LTM trace on WM maintenance.

## Experiment 1

This experiment is similar to the pilot study except for few changes. First, to reach better statistical power, it was conducted on a larger sample. Second, two consolidation interval modalities were added (600ms and 4800ms) to assess if the expected interaction between cognitive load, consolidation and item type depends on the consolidation duration.

### Method

#### Participants

To reach better statistical power, we used a *Sequential Bayes Factors Design Analysis* with maximal *n* (Schönbrodt & Wagenmakers, 2018). In this procedure, participants are sequentially added starting from a minimal *n*, and BFs are computed at each step until an a priori defined level of evidence or a defined maximal *n* is reached. We aimed at obtaining a BF_inclusion_ or BF_exclusion_ superior to 6 for each main effect and interaction in order to conclude more confidently. Therefore, we planned on recruiting a minimum of 60 participants before testing for BFs on every effect. If BFs were lower than 6 with 60 participants, one participant per experimental group was added and effects were tested again until this goal was achieved, with an upper limit set at 120 participants.

Using this method, 80 participants were recruited (25 males, 55 females). All participants respected the following criteria: aged between 18 and 30 (*M* = 21.94, *SD* = 2.90), native French speakers, normal or corrected-to-normal vision, did not self-report any history of neurological or reading problems, and provided written informed consent before taking part in the study. Participants were recruited from Lyon 2 University and received partial course credit for their participation. All participants received the same participation link and were automatically assigned to the experimental group with the least participants at the start of the experiment.

#### Material and design

The experiment was conducted online on a JATOS server (Lange et al., 2015) using jsPsych (de Leeuw, 2015). Because two consolidation modalities were added compared to the pilot experiment, a larger item pool was needed. 120 new words were selected following the same criteria as in the pilot experiment but extending the frequency lower limit to 50 occurrences per million. One pseudoword was generated for each new word using the same method described previously. In total, the material included 480 memoranda (240 words and 240 pseudowords) and 720 digits for the concurrent parity task.

As previously, we manipulated the cognitive load of the parity task (high *vs*. low) and the consolidation interval (0ms *vs*. 600ms *vs*. 2400ms *vs*. 4800ms) as within-subjects variables, and the item type (words *vs*. pseudowords) as a between-subjects variable. Two versions of the experiments were generated with random items and trials order.

#### Procedure

The experiment started with a training session followed by the experimental task, and ended with surveys on memory strategies and demographic information. The training session was divided into three phases. The first phase comprised 54 practice trials of the parity task without time limit. Accuracy was calculated, and the task had to be performed again if it did not reach 70%. The second phase corresponded to 10 example arithmetic problems that latter served as a distracting task at the end of each block. The third phase was similar to the experimental task, consisting of eight trials so that every combination of cognitive load and consolidation duration was presented. After the example of another online experiment (Loaiza et al., 2020, Experiment 3), performance on the secondary parity task was monitored during the entire experiment. If their correct-response rate reached the lower-limit of 70%, participants were reminded the importance of paying attention to this task and were warned that they had to increase their performance to prevent being sent back to the practice phase. A new performance check was done three trials latter, and participants were indeed required to complete again the parity practice phase if their performance had not increased above 70%.

The experimental task was a complex span task, in which the items to be recalled might be words or pseudowords depending on the experimental group. One experimental session consisted of 48 trials of 5 memoranda. A trial started with a fixation cross presented during 1000ms, followed by the presentation of the first item. Each item was displayed during 2000ms. Depending on the consolidation condition of the trial, the parity task was presented right after each item (0ms) or after an unfilled delay (600ms, 2400ms or 4800ms). A parity judgement was asked (“m” key for even and “q” for odd on an Azerty keyboard) for three successive digits. Depending on the cognitive load, each digit was presented for 1200ms (low cognitive load) or 600ms (high cognitive load). The presented stimuli of the trial were separated by an interstimulus interval of 150ms. Words and digits were read silently.

At the end of a trial, the word “*rappel*” (recall) was displayed, allowing the participant to recall all the target items of the trial in the original order on a response screen. The participants had to click a button to move on the next trial, with the possibility to take breaks freely before beginning the next trial. After each recall screen, an intermediate screen asked the participants to place their fingers back on the *q* and *m* keys and press the space key when ready. To increase data size for delayed recall and avoid a floor effect, the experiment was constituted of 3 blocks of 16 trials. At the end of each block, participants were required to complete an unrelated distraction task of multiplication problems (e.g., 4 × 5 = 20?) for 1 min. After this distracting task, participants performed a delayed recall in which they were invited to recall the 80 memoranda of the block in any order and without time limit. This delayed recall stopped on participant’s input, showing a break screen before continuing to the next block. After the last delayed recall, participants completed a form on maintenance strategies used, entering a use percentage for each strategy proposed. A fillable line was also added, allowing them to add a specific strategy that they used but was not listed in the form. Finally, a demographic survey was presented, after which the experiment ended.

#### Hypotheses

We expected a main effect of item type, with better recall for words than pseudowords at both immediate and delayed recall, in line with the idea that pseudowords do not benefit from LTM representations. As observed in previous studies (Barrouillet et al., 2007; Camos & Portrat, 2015; Plancher & Barrouillet, 2013), we predicted an effect of cognitive load, with a poorer recall under high than low cognitive load at immediate and delayed recall. We also expected a cognitive load × item type interaction, with a cognitive load effect for words but not for pseudowords, in line with interpretations in the literature indicating than unfamiliar material cannot be refreshed (Loaiza, Duperreault, et al., 2015; Nees et al., 2017; Ricker & Cowan, 2010; Vergauwe et al., 2014). We expected better recall performance with increased consolidation duration at immediate recall, as observed previously (Bayliss et al., 2015; De Schrijver & Barrouillet, 2017). In line with a recent finding (Cotton & Ricker, 2021), we also hypothesized increased delayed recall performance with longer consolidation intervals. Finally, if refreshing relies on WM representation stability as stated by our theoretical hypothesis, and short-term consolidation contributes to this stability, we should observe a cognitive load × consolidation × item type interaction, with a cognitive load effect for pseudowords at longer consolidation intervals but no effect at shorter consolidation times. Given that words already have stable representations, consolidation time should not moderate the effect of cognitive load on words.

### Results

As in the pilot experiment, participants with performance lower than 70% in the concurrent parity task were discarded from further analyses (22 participants excluded, final sample *n* = 58, 35 in the words group). Participants included in the analyses were sent back to practice due to parity performance less often (*M* = 0.29, *SD* = 0.56) than excluded participants (*M* = 3.91, *SD* = 3.12).

#### Main analysis

Analyses were conducted similarly to the pilot experiment. We conducted Bayesian analyses of variance (BANOVA) on correct recall percentage for immediate serial, immediate free and delayed recall scorings, using the cognitive load (high vs. low), the consolidation interval (0ms vs. 600ms vs. 2400ms vs. 4800ms) and the item type (words vs. pseudowords) as predictive variables and subjects as a random factor. To identify removable interactions, interactions that can be undone by a monotonic transformation of the measurement scale and thus give ambiguous interpretations (Wagenmakers et al., 2012), the survivability of every interaction with at least substantial evidence was tested. For that, a *logit(p)* transformation was applied on the dependent variable and the evidence supporting the interaction was calculated by comparing BFs of a model including both its main effects and the interaction, against a model including only the main effects. Interactions that did not survive this transformation were not interpreted.

**Figure 3:**
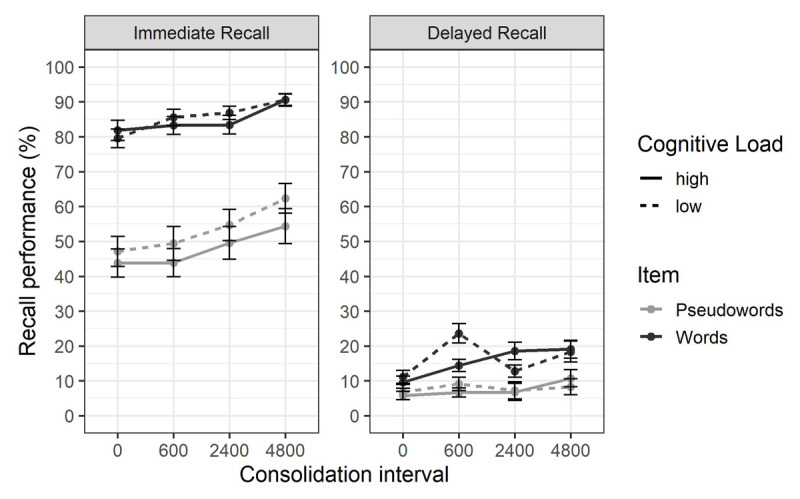
**Mean Percentage of Correct Immediate Serial Recall and Delayed Recall in Experiment 1.** Percentage of correct recall is shown according to the item type (words vs. pseudowords), the cognitive load of the concurrent processing task (high vs. low) and the consolidation interval (0ms vs. 600ms vs. 2400ms vs. 4800ms). The error bars represent the standard error of the mean.

At immediate recall (Figure 3), the best model included the main effects of item type, consolidation duration and cognitive load, and the interaction between item type and cognitive load (BF_10_ = 4.91e+28). The resulting model was similar to the one found in the pilot experiment, excepted for the addition of the interaction between item type and cognitive load. As expected, there was a main effect of item type (BF_inclusion_ = 1.92e+08), with a better recall for words (*M* = 85.19, *SD* = 11.94) than for pseudowords (*M* = 50.63, *SD* = 19.80). We found extreme evidence for a main effect of consolidation duration (BF_inclusion_ = 5.68e+08), with better recall performance as consolidation duration increases (0ms: *M* = 66.69, *SD* = 24.31; 600ms: *M* = 69.43, *SD* = 24.91; 2400ms: *M* = 72.04, *SD* = 23.05; 4800ms: *M* = 77.79, *SD* = 22.03). We also observed very strong evidence for an effect of cognitive load (BF_inclusion_ = 35.23), recall performance being better under low cognitive load (*M* = 72.83, *SD* = 21.97) than under high cognitive load (*M* = 70.14, *SD* = 24.23). Substantial evidence for an interaction between cognitive load and item type (BF_inclusion_ = 9.03) indicated that the cognitive load effect was stronger for pseudowords (+5.47) than for words (+0.86), but the *logit* comparison provided substantial evidence against this interaction (BF_01_ = 3.25). As in the pilot experiment, we found substantial evidence against the interaction between consolidation duration and cognitive load (BF_exclusion_ = 7.52) and the interaction between consolidation duration, cognitive load and item type (BF_exclusion_ = 9.62). Evidence for other interactions was inconclusive.

To evaluate if the tested factors improved item memory or order memory, the same analysis was also conducted on immediate free recall scoring (i.e., not taking into account the order of recall). The best model included the same effects than when using immediate serial recall scoring, excepted for the addition of the interaction between item type and consolidation interval (BF_10_ = 2.46e+34). However, the isolated evidence for this interaction was inconclusive (BF_inclusion_ = 2.19).

At delayed recall (Figure 3), the best model included the main effects of item type, consolidation and cognitive load, the interaction between item type and consolidation, and the interaction between consolidation and cognitive load (BF_10_ = 4.21e+07). We found strong evidence for an effect of item type (BF_inclusion_ = 21.74) with better recall for words (*M* = 15.94, *SD* = 9.60) than for pseudowords (*M* = 7.68, *SD* = 8.48). There was extreme evidence for the main effect of consolidation duration (BF_inclusion_ = 6.11e+04), reflecting worst memory performance at 0ms (*M* = 8.76, *SD* = 8.63) than at others consolidation durations (600ms: *M* = 14.60, *SD* = 11.45; 2400ms: *M* = 12.24, *SD* = 11.66; 4800ms: *M* = 15.06, *SD* = 13.89). The best model also included the interaction between consolidation and item type, suggesting a stronger effect of consolidation for words (+8.33 between 0ms and 4800ms) than for pseudowords (+3.19 between 0ms and 4800ms), but isolated evidence for this interaction was inconclusive (BF_inclusion_ = 1.68) and the *logit* comparison indicated substantial evidence against it (BF_01_ = 3.43). We observed substantial evidence against an effect of cognitive load (BF_exclusion_ = 6.41). There was extreme evidence for an interaction between cognitive load and consolidation (BF_inclusion_ = 141.02). As this interaction was not expected, unplanned post-hoc comparisons were conducted. Bayesian *t* tests on delayed recall performance between high and low cognitive load conditions were done at each consolidation interval. Those analyses revealed moderate evidence against an effect of cognitive load at 0ms (BF_01_ = 4.15) and 4800ms (BF_01_ = 5.04) consolidation durations. There was extreme evidence supporting the cognitive load effect at 600ms consolidation interval (BF_10_ = 214.04). Evidence was inconclusive at 2400ms (BF_10_ = 1.20). Together, these comparisons indicate a cognitive load effect at delayed recall with a 600ms consolidation interval, but not with 0ms nor 4800ms consolidation duration. However, the *logit* comparison indicated substantial evidence against this interaction (BF_01_ = 4.65). Contrary to immediate recall, there was substantial evidence against the interaction between cognitive load and item type (BF_exclusion_ = 6.37). Evidence for other interactions was inconclusive.

#### Cumulated maintenance time analysis

As planned, we conducted supplementary analyses on the cumulated maintenance time. The objective was to test if using cumulated maintenance time better predicted recall performance than a model distinguishing all factors manipulated in this experiment. On both immediate and delayed recall scorings, we compared the best model identified by the main analyses to a model including item type and cumulated maintenance time. Cumulated maintenance time was calculated for each item as the time between presentation offset and the recall screen, which took into account its serial position, and the cognitive load and consolidation conditions. Recall performance was then averaged by item type and cumulated maintenance time value, as a strong effect of item type was expected and could have biased results in favor of the full model if not included. As suggested by previous work (Jarjat et al., 2018, 2020; Loaiza et al., 2022), a logarithmic relation was expected between cumulated maintenance time and recall performance. Therefore, for both immediate and delayed recall scorings, a first analysis was conducted to determine if a linear or logarithmic relation was best suited for the cumulated maintenance time model, which was then compared to the full model previously identified.

**Figure 4:**
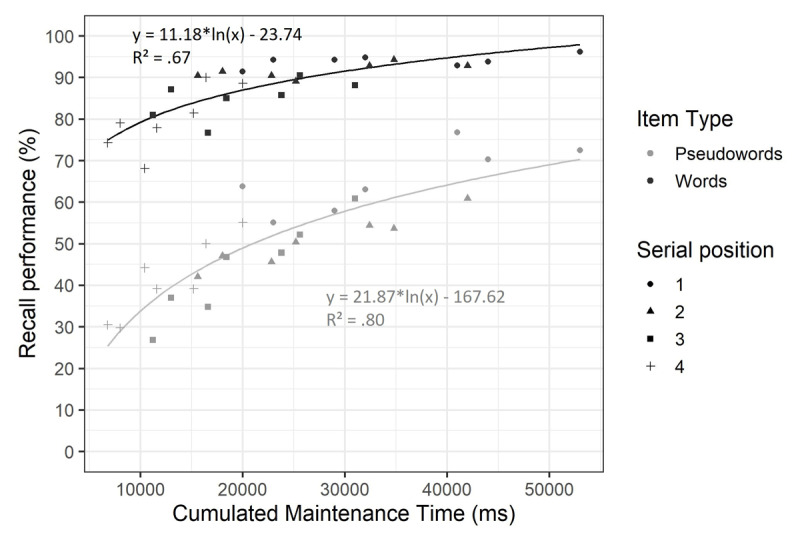
**Mean percentage of correct immediate serial recall by cumulated maintenance time in Experiment 1.** Percentage of correct recall is shown according to item type (words vs. pseudowords), serial position (1 to 4) and cumulated maintenance time.

At immediate recall (Figure 4), a strong recency effect was observed, particularly for pseudowords. Thus, it seemed better suited to remove the last item of each trial from the data for analyses on immediate serial recall. As observed in the literature, there was extreme evidence in favor of the logarithmic model over the linear model (BF = 1.45e+08), which was therefore kept for following comparisons. The previously identified best model included the main effects of item type, consolidation duration and cognitive load, and the interaction between item type and cognitive load. There was extreme evidence in favor of the model including cumulated maintenance time and item type (BF = 8.01e+62) compared to this full model. However, because cumulated maintenance time includes items’ serial position, which is not considered in the full model, we conducted an additional unplanned analysis after adding serial position as a predictor in both the cumulated free time and the full model. This comparison yielded inconclusive evidence (BF = 1.23), suggesting that the cumulated maintenance time model’s strength may have been driven by the inclusion of serial position into its calculation.

**Figure 5:**
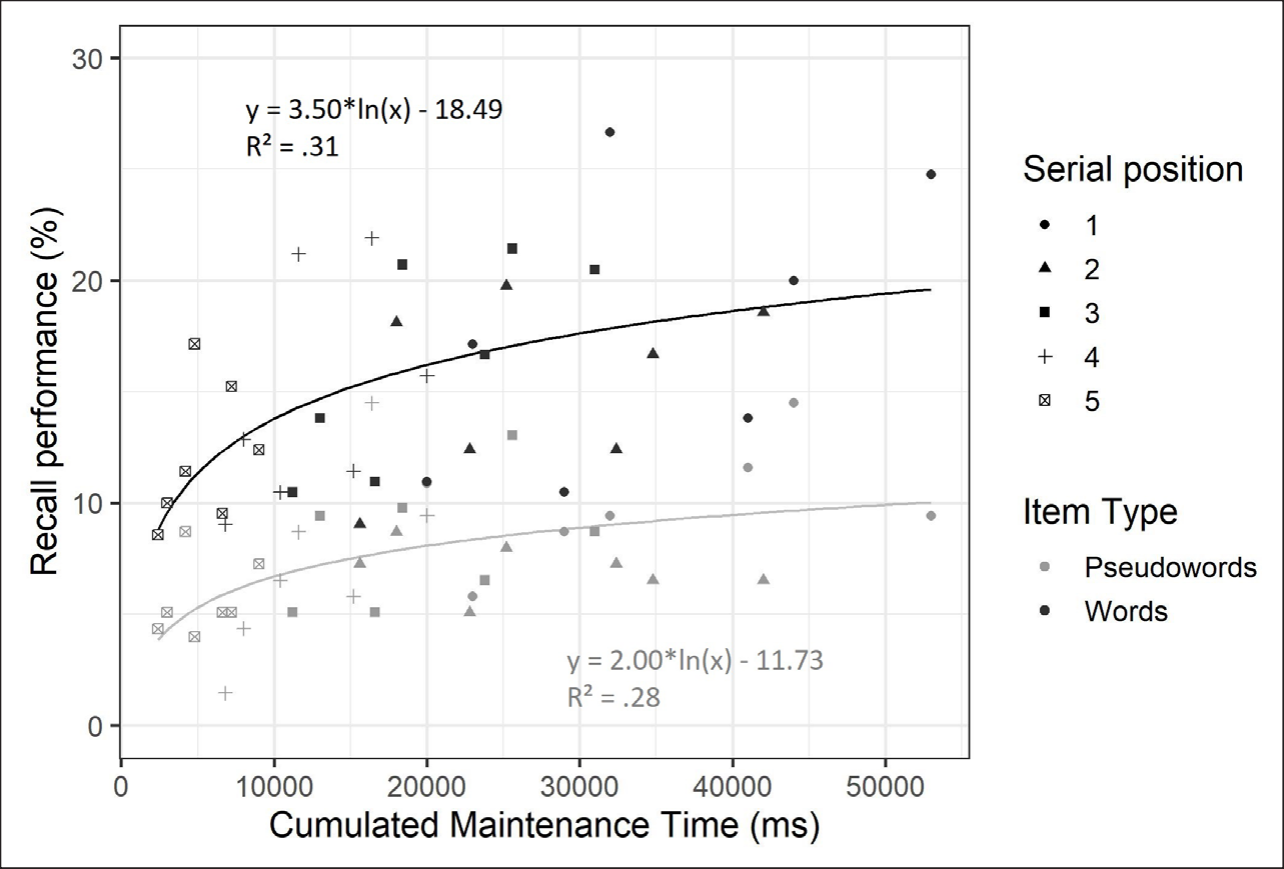
**Mean percentage of correct delayed recall by cumulated maintenance time in Experiment 1.** Percentage of correct recall is shown according to item type (words vs. pseudowords), serial position (1 to 5) and cumulated maintenance time.

The same analysis was conducted on delayed recall scoring (Figure 5). There was strong evidence favoring the logarithmic model over the linear model (BF = 12.15). The best model identified in the main analysis included the main effects of item type, consolidation duration and cognitive load, the interaction between item type and consolidation and the interaction between consolidation and cognitive load. Again, this model was compared to a model including the cumulated maintenance time and item type, revealing extreme evidence in favor of the full model (BF = 4.10e+15).

#### Conditional recall analysis

To better understand what stability of WM representations means, we planned on analyzing a conditional immediate recall scoring based on previous work (Jarjat et al., 2018, 2020). This conditional scoring only considers items that are not latter recalled in the delayed recall test, to remove the participation of LTM in WM performance. A Bayesian ANOVA was conducted on this scoring using the same design as in the main analysis. The best model was the same as in the main analysis, including the effects of item type, consolidation duration and cognitive load, and the interaction between item type and cognitive load (BF_10_ = 1.29e+27).

#### Strategies

**Figure 6:**
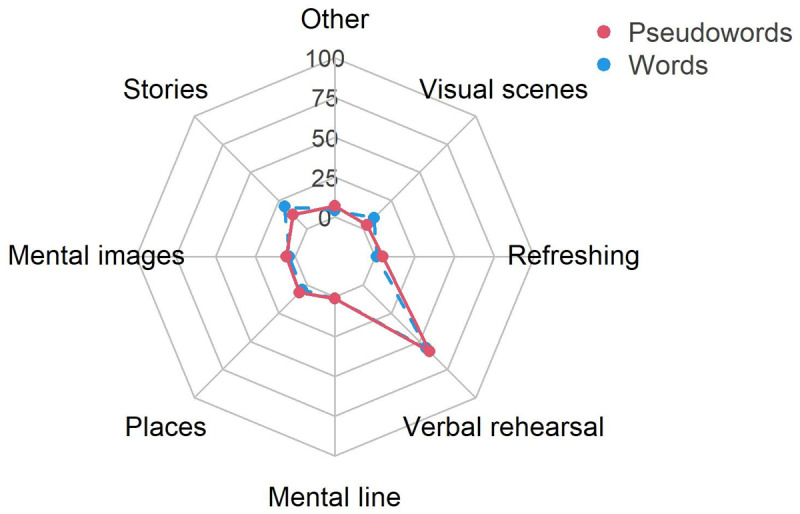
Mean percentage of strategy use by item type group in Experiment 1.

By design, data collected through the strategies survey was unsuited for statistical analyses as it consisted mainly of between one and three values for each participant and only zeros for every other strategy. Nonetheless, descriptive analysis could provide some insight in possible differences when the task was completed with words or pseudowords (Figure 6). First, we observed that verbal rehearsal was overwhelmingly more used that any other strategy (words: *M* = 56.32; pseudowords: *M* = 60.15). There seemed to be no notable difference between the two participants groups, except for a slight increase in use of stories (words: *M* = 19.95; pseudowords: *M* = 9.82) and visual scenes (words: *M* = 9.07; pseudowords: *M* = 3.32) when maintaining words. Reported use of attentional refreshing was low (words: *M* = 1.30; pseudowords: *M* = 5.62).

### Discussion

The main objective of this experiment was to evaluate if adding free time for short-term consolidation improved refreshing of a novel material, pseudowords. Contrary to our hypotheses we observed that increasing the cognitive load of the concurrent processing task impaired immediate serial recall performance of pseudowords, and this effect did not interact with manipulations on short-term consolidation duration. Thus, our results suggest that pseudowords can be refreshed and that short-term consolidation does not affect refreshing availability.

Consistent with previous literature, we found that increasing consolidation duration improved recall performance at short-term (Bayliss et al., 2015; De Schrijver & Barrouillet, 2017; Ricker & Cowan, 2014). In addition and in line with a recent study using delayed recognition tests (Cotton & Ricker, 2021), we also observed a consolidation benefit at long-term for both words and pseudowords. They also found evidence that consolidation affected words and pseudowords equally. Similarly, we found no evidence that the effect of consolidation was different between words and pseudowords, suggesting that short-term consolidation does not depend on the LTM content. The absence of interaction between cognitive load and consolidation duration at short-term has been observed before, and interpreted sometimes as the independence of the two mechanisms (Bayliss et al., 2015) or their substitutability (De Schrijver & Barrouillet, 2017) because of the similar benefit of adding more time for either consolidation or maintenance. Our results replicated this absence of interaction at short-term and revealed different patterns for consolidation and cognitive load manipulations at long-term. First, consolidation improved delayed recall performance while the manipulation on cognitive load had more ambiguous effects. Moreover, analyses on cumulated maintenance time indicated that only considering the delay between memoranda presentation and their recall is worst at predicting delayed recall performance than accounting for both cognitive load and consolidation independently. Together, these results provide supplementary evidence for the independence of consolidation and refreshing.

It has been suggested that cumulated free time could be a better factor to predict delayed recall performance than various predictors, such as cognitive load or number of distractors (Jarjat et al., 2018, 2020; Loaiza et al., 2022; Souza & Oberauer, 2017). Results at immediate recall are more ambiguous. One study (De Schrijver & Barrouillet, 2017) argued, using a different manipulation, that free time before or after the distracting task was substitutable (i.e., consolidation duration and cognitive load) and improved short-term recall performance. Conversely, the study of Souza and Oberauer (2017) observed that immediate recall performance in a slow span condition (i.e., with unfilled intervals between memoranda) was at the same level than in a simple span condition, suggesting that increasing maintenance time did not improve short-term recall. By conjointly manipulating cognitive load and consolidation and using a Bayesian framework, our experiment provided a good opportunity to investigate this hypothesis more directly by comparing its predictive power to that of considering the factors independently. While cumulated maintenance time seemed to be able to better predict immediate serial recall performance than a full model at first glance, this effect seemed mainly driven by the addition of serial position into the calculation and thus additional investigations may be necessary. At delayed recall, our results strongly favored a full model over cumulated maintenance time contrary to what has been suggested in the literature. However, we note that contrary to previous work, we did not manipulate the number of distractors but did modulate consolidation time. Therefore, it is possible that cumulated free time is a better predictor of delayed recall than cognitive load and number of distractors, but cannot account for consolidation duration that has a distinct effect.

## Experiment 2

The pilot and the first experiment aimed at testing how consolidating WM representations can affect refreshing of novel material. The purpose of this second experiment was to investigate the stability of WM representations through another manipulation, the repetition of memoranda. We manipulated the stability of pseudowords by repeating them throughout the experiment to observe its potential effect on their maintenance. In a complex span task using words and pseudowords, half the memoranda were repeated three times in different trials to observe the changes induced in the effect of cognitive load and its interaction with item type.

### Method

#### Participants

Using the same method and criteria as in Experiment 1, 84 new participants were recruited for this experiment (18 males, 64 females) with a mean age of 22.27 (*SD* = 2.88).

#### Material and design

The experiment was conducted online on a JATOS server (Lange et al., 2015) using jsPsych (de Leeuw, 2015). 240 items (120 words and 120 pseudowords) and 720 digits were randomly selected from the material of experiment 1. Half the memoranda were presented three times in different trials, while the other half was presented once. Trials containing first-presented items and trials containing repeated items were intermixed throughout the entire experiment. To ensure this, the alternation between trials containing only-once-presented items and trials containing repeated items was fixed, with one once-presented-items trial every three repeated-items trials. To avoid associations between repeated items, each repeated item was presented at a different serial position at each of its repetition and was not coupled with the same items more than two times.

We manipulated the cognitive load of the concurrent task (high vs. low) and the total number of item presentations (1 vs. 3) as within-subjects variables and the item type as a between-subjects variable. Two versions of the experiments were generated with random items and trials order.

#### Procedure

The experiment started with a training session followed by the experimental task, and ended with surveys on memory strategies and demographic information. The training session was divided into three phases. The first phase comprised 54 practice trials of the parity task without time limit. Accuracy was calculated, and the task had to be performed again if it did not reach 70%. The second phase corresponded to 10 example arithmetic problems that latter served as a distracting task at the end of each block. The third phase was similar to the experimental task, consisting of four trials with two trials at both low and high cognitive load. As in experiment 1, performance on the secondary parity task was monitored during the entire experiment and participants were sent back to the practice phase if they were underperforming.

The experimental task was a complex span task, in which the items to be recalled might be words or pseudowords depending on the experimental group. One experimental session consisted of 48 trials of 5 memoranda, separated into 2 blocks of 24 trials. A trial started with a fixation cross presented for 1000ms, followed by the presentation of the first item. Each item was displayed for 2000ms. The parity task was presented right after each item. A parity judgement was asked (“m” key for even and “q” for odd on an Azerty keyboard) for three successive digits. Depending on the cognitive load, the digits were presented for 1200ms (low cognitive load) or 600ms (high cognitive load) per digit. The presented stimuli of the trial were separated by an interstimulus interval of 150ms. Half the items were presented three times across the experiment, and the other half once. Words and digits were read silently.

At the end of a trial, the word “*rappel*” (recall) was displayed, allowing participants to recall all the target items of the trial in the original order on a response screen. Participants had to click a button to move on the next trial, with the possibility to take breaks freely before beginning the next trial. After each recall screen, an intermediate screen asked the participants to place their fingers back on the *q* and *m* keys and press the space key when ready. At the end of each block, participants were required to complete an unrelated distraction task of multiplication problems (e.g., 4 × 5 = 20?) for 1 min. After this distracting task, participants performed a delayed recall in which they were invited to recall the 60 target items of the block in any order and without time limit. This delayed recall test stopped after the participants indicated that they could not recall any more items, displaying a break screen before continuing to the next block. As in experiment 1, the last delayed recall was followed by a maintenance strategies form and a demographic survey, after what the experiment ended.

##### Hypotheses

As in experiment 1 and for the same reasons, we expected an effect of item type, cognitive load, and a cognitive load × item type interaction. We also predicted an increase in performance with the number of item presentations at both immediate and delayed recall, indicating an increase in long-term traces’ strength across presentations. If our theoretical hypothesis is true, a cognitive load effect should be observed for repeated pseudowords but not pseudowords presented once, reflecting that the pseudowords repetition created a LTM trace that made them more stable in WM and allowed their refreshing.

### Results

As in the pilot and Experiment 1, the recall score included a tolerance of one mistake on each item, and participants with performance lower than 70% in the parity task were discarded (final *n* = 69, 33 in the words group). As in Experiment 1, included participants were sent back to practice less often (*M* = 0.37, *SD* = 0.72) than excluded participants (*M* = 3.63, *SD* = 2.63).

#### Main analyses

Bayesian analyses of variance were conducted at immediate and delayed recall on mean correct recall percentage, using cognitive load, number of presentations and item type as predictive factors and subjects as a random variable. Each combination of main effects and interactions was tested against the null model (including only a random effect of subjects). BF_inclusion_ or BF_exclusion_ was calculated on matching models for each effect. As in Experiment 1, default priors provided by the BayesFactor package were used. Because of the design used, the number of presentations variable had three levels at immediate recall (1 vs. 2 vs. 3) but only two levels at delayed recall (1 vs. 3). As in Experiment 1, survivability of relevant interactions to a monotonic transformation of the measurement scale was tested using the same method.

**Figure 7:**
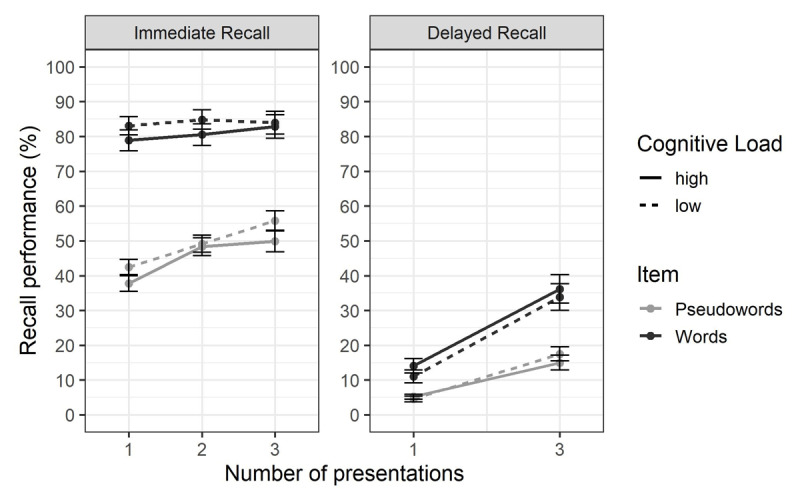
**Mean Percentage of Correct Immediate Serial Recall and Delayed Recall in Experiment 2.** Percentage of correct recall is shown according to the item type (words vs. pseudowords), the cognitive load of the concurrent processing task (high vs. low) and the number of presentations (immediate recall: 1 vs. 2 vs. 3; delayed recall: 1 vs. 3). The error bars represent the standard error of the mean.

At immediate recall (Figure 7), the best model included the effects of item type, number of presentations and cognitive load, and the interaction between item type and number of presentations (BF_10_ = 4.71e+28). There was extreme evidence for the effect of item type (BF_inclusion_ = 3.15e+11), with better recall for words (*M* = 82.01, *SD* = 15.80) than for pseudowords (*M* = 45.44, *SD* = 13.29). We found extreme evidence for the main effect of cognitive load (BF_inclusion_ = 460.59), with better performance under low (*M* = 64.81, *SD* = 23.16) than high (*M* = 61.05, *SD* = 24.07) cognitive load. We found moderate evidence for an absence of interaction between cognitive load and item type (BF_exclusion_ = 6.45). We also observed extreme evidence for an effect of the number of presentations (BF_inclusion_ = 2.48+10), with better recall for items presented 3 times (*M* = 67.44, *SD* = 23.29) than items presented twice (*M* = 64.98, *SD* = 22.49) and once (*M* = 59.65, *SD* = 24.94). This effect appeared stronger for pseudowords (+12.73 between 1 and 3 presentations) than for words (+2.40 between 1 and 3 presentations), as shown by the extreme evidence for the interaction between item type and number of presentation (BF_inclusion_ = 2.67e+04), but this interaction did not survive the *logit* transformation (BF_01_ = 4.92). There was strong evidence against an interaction between cognitive load and number of presentations (BF_exclusion_ = 13.16). We hypothesized a stronger cognitive load effect for repeated pseudowords than for presented-once pseudowords, but this hypothesis relied on the belief of a difference in the cognitive load effect between words and pseudowords before the consideration of repeated items. As our results showed evidence for absence of interactions between cognitive load and item type, and between cognitive load and number of presentations, we considered this hypothesis no longer relevant, even if the evidence for the interaction between the three factors was inconclusive (BF_exclusion_ = 1.74). To further confirm this, we conducted an unplanned *t* test, comparing the effect of cognitive load on immediate recall performance between presented-once pseudowords and pseudowords repeated three times. This comparison showed moderate evidence against a difference (BF_01_ = 5.00).

Using immediate free recall scoring, the best model included the same effects and interaction than with immediate serial recall scoring (BF_10_ = 9.43e+50).

At delayed recall (Figure 7), the best model included the effects of item type, number of presentations and the interaction between item type and number of presentations (BF_10_ = 4.10e+32). There was extreme evidence for the effect of item type (BF_inclusion_ = 3.60e+03), with a better recall for words (*M* = 29.38, *SD* = 17.63) than for pseudowords (*M* = 13.40, *SD* = 9.09). We found extreme evidence for an effect of the number of presentations (BF_inclusion_ = 1.18e+26) with better recall for repeated items (*M* = 25.22, *SD* = 19.15) than for items presented once (*M* = 16.87, *SD* = 12.90). This effect seemed larger for words (+11.24) than for pseudowords (+5.69) as indicated by the extreme evidence in favor of the interaction between item type and number of presentations (BF_inclusion_ = 1.47e+03), but again the interaction did not survive the *logit* transformation (BF_01_ = 5.04). However, we observed substantial evidence against an effect of cognitive load (BF_exclusion_ = 6.28). There was moderate evidence against an interaction between cognitive load and number of presentations (BF_exclusion_ = 4.20) and an interaction between cognitive load, number of presentations and item type (BF_exclusion_ = 3.41). Evidence regarding the interaction between cognitive load and item type was inconclusive (BF_exclusion_ = 2.09).

#### Conditional recall analysis

As in Experiment 1, we conducted a Bayesian analysis of variance on a conditional immediate recall scoring, including only items not recalled in delayed recall tests. The best model was the same than in the main analysis, including the main effects of item type, number of presentations and cognitive load, and the interaction between item type and number of presentations (BF_10_ = 6.98e+25).

#### Strategies

**Figure 8:**
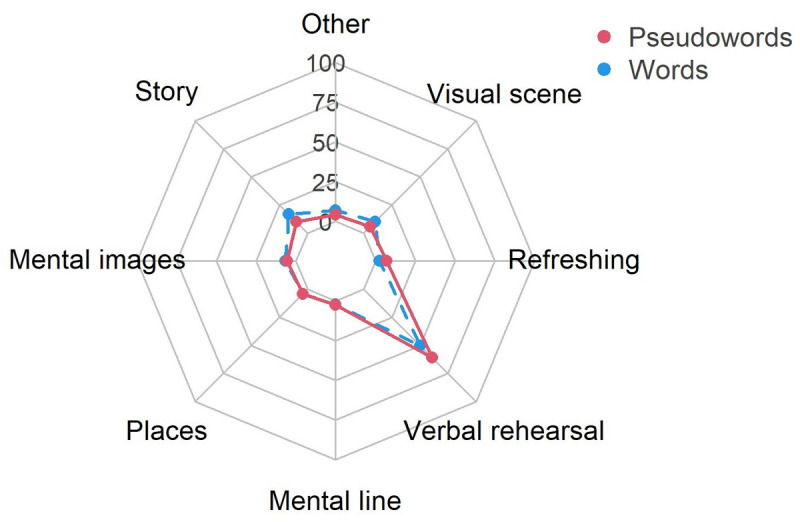
Mean percentage of strategy use by item type group in Experiment 2.

For the same reasons as in Experiment 1, only data descriptions are presented here (Figure 8). Similar results were observed. We observed that verbal rehearsal was overwhelmingly more used that any other strategy (words: *M* = 50.27; pseudowords: *M* = 60.64). Stories (words: *M* = 17.03; pseudowords: *M* = 10.05) and visual scenes (words: *M* = 10.15; pseudowords: *M* = 5.64) were more used when maintaining words than pseudowords, and reported use of refreshing was low (words: *M* = 2.67; pseudowords: *M* = 6.94).

### Discussion

The purpose of this second experiment was to investigate the effect of representations’ stability on WM maintenance through another manipulation, the repeated presentations of items. As in Experiment 1, we observed an effect of cognitive load on immediate recall performance even for pseudowords. This result reinforces our conclusions in Experiment 1, by showing no evidence that pseudowords cannot be refreshed.

We observed a strong effect of repeating items on memory performance, at both immediate and delayed recall. However, we found no evidence that repeating items modulated the effect of cognitive load, suggesting that it did not affect refreshing efficiency.

## General discussion

The objective of the present study was to test a new hypothesis regarding refreshing functioning, stating that refreshing efficiency depends on WM representations’ stability. Through two experiments, we manipulated WM stability for words and pseudowords using short-term consolidation (Experiment 1) or multiple presentations of memoranda (Experiment 2) to evaluate if it would affect refreshing.

### Attentional maintenance in working memory

Previous literature has indicated that attentional refreshing use could depend on the status of preexisting LTM traces. It has been shown that the benefit of complex span tasks over simple span tasks on long-term recall was observed for words but not pseudowords (Loaiza, Duperreault, et al., 2015). Similarly, other studies suggested that some material could not be refreshed (Nees et al., 2017; Ricker & Cowan, 2010; Vergauwe et al., 2014). Together, these results seem to indicate that refreshing cannot be used to maintain unfamiliar or novel material. Conversely, it has also been observed that manipulations on refreshing availability (i.e., cognitive load) were not modulated by the use of semantic cues over phonological cues (Loaiza & Camos, 2018), nor effects of word frequency and lexicality (Camos, Mora, et al., 2018). These latter studies indicate that refreshing functioning does not seem to depend on semantic content in LTM. Therefore, we suggested that WM representations’ stability could determine the ability to use refreshing, thus explaining the results observed in the literature.

In this series of experiments, including the pilot experiment, we consistently observed a cognitive load effect on immediate recall performance and evidence that this effect was not different between words and pseudowords. As manipulations on cognitive load have been proposed to reflect modulation of refreshing availability^1^ (Barrouillet et al., 2004; Camos et al., 2009; Camos, Johnson, et al., 2018), our results suggest that like words, pseudowords could be maintained using refreshing. These results replicate a previous study (Camos, Mora, et al., 2018) which observed that item lexicality did not modulate the effect of cognitive load. We expanded it by showing that the effect of cognitive load on immediate recall for both words and pseudowords was still observed after removing items correctly recalled at long-term, indicating that even pseudowords that did not benefit from a successful LTM encoding could be refreshed. This lead us to draw the same conclusions than Camos, Mora and colleagues (2018) against views of refreshing as a reconstruction from LTM (Barrouillet & Camos, 2015) or retrieval of semantic information from LTM (Loaiza, Duperreault, et al., 2015; McCabe, 2008).

A possible explanation could have been that LTM improves the stability of representations in WM, participating in creating a refreshable WM representation, without any direct interaction between refreshing and LTM (for a similar proposition, see Camos, Johnson, et al., 2018). However, we found no evidence that the effect of cognitive load was affected by manipulations on short-term consolidation (Experiment 1) nor number of memoranda presentations (Experiment 2), even while they improved immediate recall performance. These findings do not support the idea that refreshing improves with WM representations stability. As pseudowords appeared to be refreshable before our manipulations on stability, it could be argued that it is still possible that stability participates in creating a refreshable representation in WM but does not further facilitate refreshing past this point, leading to the same pattern of results. However, this view would implicate that pseudowords presented once, not benefiting from added consolidation time and not having strong enough episodic trace that they can be recalled latter (i.e., conditional immediate recall scoring) still have stable WM representations that allow their refreshing, which seems implausible. Thus, we conclude that WM representations’ stability does not seem to be a determining factor for the use of attentional refreshing.

### Effect of working memory on long-term memory

It has been observed previously that increasing the attentional demand during the WM task impaired long-term recall performance (Camos & Portrat, 2015; Jarjat et al., 2018, 2020), interpreted as a role of refreshing in promoting LTM traces. However, in the experiments that we conducted including the pilot, we consistently found evidence for an absence of effect of cognitive load on delayed recall performance. In a recent preprint, Loaiza and colleagues (2022) conducted a series of six experiments while manipulating, among other factors, the cognitive load. In four out of the six experiments, they found evidence against or no evidence for an effect of cognitive load on delayed recall. One possibility to explain this discrepancy is that the effect of cognitive load on delayed recall that has been observed in the literature did not reflect the action of refreshing, but of another mechanism that was also impaired by attentional manipulations. A previous study using explicit instructions concluded that refreshing only serves to preserve memory performance at short-term and does not affect long-term recall, while elaboration promotes LTM formation (Bartsch et al., 2018). Following this viewpoint, the pattern of results observed in our experiments could be explained by the long presentation time used for memoranda (2s), originally chosen to allow correct encoding of pseudowords. This may have allowed elaborative mechanisms to take place during presentation time rather than during the processing task, leaving our manipulations of cognitive load to only affect attentional refreshing and therefore only short-term recall performance. Another possibility is that the other factors manipulated (short-term consolidation in Experiment 1 and pilot experiment; number of presentations in Experiment 2) affected memory in a way that nullified the benefit of refreshing on LTM. Because both consolidation and item repetition improved long-term recall, they may have promoted LTM formation the same way refreshing usually does, leaving no room for the added use of refreshing. This seem unlikely, both because it does not really explain the inconsistent effect of cognitive load in the study of Loaiza and colleagues (2022), and because we would have expected an effect of cognitive load in the conditions without added consolidation time and with presented-once memoranda in our experiments. Further studies will be needed to identify the boundaries conditions for observing an effect of cognitive load on LTM, as some studies have started to investigate on short-term recall (Ricker & Vergauwe, 2022).

Attentional refreshing is not the only way for WM to promote LTM formation. Experiment 1 showed that short-term consolidation improved delayed recall, confirming what has recently been observed with long-term recognition (Cotton & Ricker, 2021). In their study, Cotton and Ricker discussed that they could not rule out a role of refreshing in the effect of consolidation on LTM. As discussed previously, we observed an absence of interaction between consolidation duration and cognitive load at immediate recall, and distinct pattern between the two factors at delayed recall. Additionally, merging consolidation and cognitive load (i.e., cumulated maintenance time) was worst at predicting delayed recall performance than considering both factors distinctively. Together, these results do not support an action of refreshing during consolidation periods. Another explanation of the effect of consolidation on LTM could be the involvement of elaborative strategies during this interval. Cotton and Ricker’s (2021) study provided arguments against this interpretation, as their design did not make such strategies relevant but still observed a long-term benefit of consolidation. Additionally, Bartsch and colleagues (2018) observed that elaboration improved long-term but not short-term performance. Therefore, as consolidation improved immediate recall in both our experiments and in previous work, explaining the long-term effect of consolidation by the action of elaboration would require that participants engaged in both short-term consolidation and elaborative strategies during short-term consolidation time-windows to justify an effect at both immediate and delayed recall. Supporting this, we note that memory performance steadily improved immediate recall performance the longer the duration of consolidation, while in contrast delayed recall performance benefited from the addition of a consolidation interval but this improvement was not larger when further increasing the duration of this interval. Thus, it could be that adding a consolidation interval provided opportunity for elaborative strategies that improved delayed recall, and that further increasing the duration only led to additional consolidation that improved immediate recall. However, few participants reported the use of elaborative strategies in Experiment 1. Moreover, their use was no larger than in Experiment 2 where no additional consolidation time was added, providing arguments against this explanation. Therefore, in light of the current results, we think an effect of short-term consolidation in promoting long-term recall seems the most plausible hypothesis to explain the observed long-term benefit, rather than the implication of a concurrent and independent mechanism. Further investigating short-term consolidation appears to be a promising lead in better understanding the links between WM and LTM.

In Experiment 2, we observed that repeating memoranda multiple times in the experiment improved both their short-term and long-term recall. This manipulation was introduced as a way to improve WM representations’ stability, with the idea that it would provide items some grounding in LTM, particularly for pseudowords. Its benefit for both immediate and delayed recalls was therefore not surprising, but discussing what could explain this effect still appears interesting. It could have been though that the effect of repetition reflected a testing effect, the improvement of memory performance with each recall (Bjork, 1975), with repeated items being tested multiple times in immediate recall tests and thus being better recalled at subsequent immediate and delayed recall tests. However, this would lead us to expect that items correctly recalled are more likely to be recalled again latter. As low cognitive load items were more recalled than high cognitive load items at short-term but not at long-term, it does not support this interpretation. Similarly, an explanation of the effect of repetition by the number of refreshing opportunities seems implausible, as we would have expected an increased effect of cognitive load at least for repeated items that would reflect this action of refreshing. Thus, we consider that two interpretations could explain the observed effects of the number of presentations. First, it is possible that each presentation of a repeated memoranda provided a new opportunity for short-term consolidation. While no specific consolidation window was present in Experiment 2, it could still be implemented during the item presentation and the following interstimulus interval (2000ms + 150ms). Thus, the cumulation of multiple consolidation opportunities would increase memory performance for both short-term and long-term tests. However, we feel this interpretation alone lacks a reliable way of explaining how a previously consolidated item is more easily maintained the next time it is presented. A second interpretation is that this effect could be due to the contribution of LTM during recall. A recent study (Bartsch & Shepherdson, 2021) showed that workload can be outsourced to LTM to optimize performance by contributing to retrieval during WM recall tests. As its action is constricted to the recall phase, no effect on WM maintenance mechanisms would be expected, consistent with the absence of interaction between cognitive load and number of presentations that we observed. This interpretation could explain the benefit to memory at both short and long-term, the absence of interaction with cognitive load, and can be considered alone or conjointly to the previous one regarding consolidation opportunities. Our analyses on conditional immediate recall do not support this hypothesis, as the effect of the number of presentations was still observed when including only items not latter recalled at delayed recall tests. However, it cannot completely rule it out either, as it is reasonable to consider that a memorandum not being correctly recalled in free delayed recall tests does not prove its LTM traces was not strengthened, thus using cued recall or recognition tests may be more suited to further discuss this point. Nonetheless, manipulating the number of presentations in future studies, as a way to gradually improve LTM storage of information, can be a useful tool to investigate how other manipulated factors of interest interact with the content of LTM, as we did in Experiment 2.

### Are working memory and long-term memory distinct?

There is a long-lasting debate around the question of whether WM and LTM are two distinct memory systems, or if there is a single memory system responsible for both short- and long-term storage (for a current view on this debate, see for instance Cowan, 2019; Norris, 2017). For instance, one of the prominent theories of the latter view is the *Embedded-Processes Model* (Cowan, 1999, 2019). In this model, short-term memory results from the combination of information held in the focus of attention, the content of activated LTM (i.e., a subset of LTM readily available) and a rapid new learning of some aspects of the presented information (e.g., the serial positions of list items) into activated LTM that can be used in the trial. To explain various experimental results regarding maintenance time, it is proposed that free time during trials is used to construct more stable long-term representations and a better activated LTM for the task (Cowan, 2019). However, we believe our results provide arguments supporting a distinction between WM and LTM. In particular, different results in the present study suggest that refreshing, a WM mechanism, acts independently from LTM.

We observed that the effect of cognitive load was not modulated by items’ lexicality, indicating that refreshing can operate similarly on words that have a previous LTM content and on pseudowords that do not. Additionally, we note that the manipulations on short-term consolidation and the number of presentations both improved delayed recall, which can indicate that they strengthened LTM representations. Still, with or without this improved LTM consolidation, attentional manipulations continued to affect short-term recall without any variation, as suggested by the absence of interactions between cognitive load and other factors. Thus, as discussed previously, refreshing does not seem to be affected by LTM content.

On the other hand, we found no evidence that WM maintenance promoted LTM formation. Cognitive load only affected immediate recall and not delayed recall in our experiments, in line with some previous work that found a similar result (Loaiza et al., 2022) or that concluded that refreshing does not affect LTM (Bartsch et al., 2018). Additionally, merging all free time into a single factor (cumulated maintenance time) was not performant in predicting delayed recall performance, which do not suggest that maintenance time in WM promoted LTM formation. In light of these results, it does not appear that refreshing participated in creating LTM traces.

Together, these results point toward the existence of an attentional mechanism that affects short-term recall, but that does not promote LTM formation and in turn is not affected by the LTM content. Overall, our results support the view that LTM contributes to WM, as the advantage of words over pseudowords and the effect of the number of presentations seem to suggest, but the two systems still appear to be distinct.

## Conclusion

We provided additional arguments against a role of LTM content in the functioning of attentional refreshing. We considered an alternative explanation that refreshing depends on WM representations’ stability, but found that it did not seem to affect refreshing efficiency either. Additionally, we investigated the effect of WM on LTM, and consistently observed that cognitive load did not affect delayed recall, contrary to previous literature. Conversely, added time for short-term consolidation and repeating items multiple times both improved short-term and long-term memory performance. We conclude that identifying what conditions the use of refreshing and whether refreshing affects LTM require further investigations.

## Data Accessibility Statement

Experiment files, materials, analysis scripts and data for the experiments are available at the Open Science Framework: https://osf.io/gctkx/. The preregistered version of this study can be found at https://osf.io/dtfrw.

## Footnotes

Alternative views applicable to complex span tasks exist, for instance describing a WM resources pool that depletes with encoding and recovers over time (Popov et al., 2019; Popov & Reder, 2020) or a response preparation process that conflicts with concurrent processing (Joseph & Morey, 2021).
